# The relation of high fat diet, metabolic disturbances and brain oxidative dysfunction: modulation by hydroxy citric acid

**DOI:** 10.1186/1476-511X-10-74

**Published:** 2011-05-14

**Authors:** Kamal A Amin, Hamdy H Kamel, Mohamed A Abd Eltawab

**Affiliations:** 1 Biochemistry Dept, Faculty of Vete. Medicine, Beni-Suef University, Beni-Suef, Egypt; 2 Clinical pathology Dept, Faculty of Vete. Medicine, Beni-Suef University, Beni-Suef, Egypt

**Keywords:** High fat diet, metabolic disturbances, BChE, brain oxidative stress, *G. Cambogia*

## Abstract

**Aims:**

This study aimed to examine the effect of high fat diet (HFD) to modulate brain dysfunction, and understand the linkages between obesity, metabolic disturbances and the brain oxidative stress (BOS) dysfunction and modulation with hydroxyl citric acid of *G. Cambogia*.

**Methods:**

Rats were divided into 3 groups; 1^st ^control, maintained on standard normal rat chow diet, 2^nd ^HFD, maintained on high fat diet along 12 week and 3^rd ^HFD+G, administered *G. Cambogia *for 4 weeks and each group include 8 rats. Blood, brain and abdominal fat were collected for biochemical measurements.

**Results:**

HFD group showed significant increase in energy intake, final BW and BW gain. Also significant increase in weight of abdominal fat in HFD group. HFD induce metabolic disturbance through increasing the lipid profile (LDL, TG, TC), γGT and α-amylase activity, uric acid level and hyperglycemia, while decreasing creatine kinase (CK) activity.

These changes associated with lowering in brain nitric oxide (NO) level and rising in serum butyrylcholinesterase (BChE), brain catalase activity and MDA levels as oxidative stress markers. These alterations improved by *G. Cambogia *that decrease BOS and increased NO level.

**Conclusions:**

Rats fed HFD showed, metabolic disturbances produce hyperglycemia, hypertriglyceridemia, hypercholesterolemia and increased LDL associated with increased BOS. Involvement of BuChE, NO and oxidative stress associated with metabolic disturbances in the pathophysiological progression in brain, suggesting association between obesity, metabolic disorders and brain alteration while, using *G. Cambogia*, ameliorate the damaging effects of the HFD via lowering feed intake and BOS.

## 1- Introduction

The brain is exceptionally susceptible to oxidative stress that may be caused by high fat diet and xenobiotics such as ethanol. HFD and alcohol metabolism is accompanied by enhanced free radicals formation and a decrease in antioxidant abilities. Prolonged HFD promotes a variety of morbidity factors although experimental evidence for short-term western diet (WD) mediating brain dysfunction remains to be elucidated. The prevalence of central obesity among Egyptian adults was 24.1% and 28.7% based on the waist circumference and waist-to-hip ratio indicators respectively [1].

It has been shown that the disturbances originated by high-fat feeding closely resemble the metabolic disturbances observed in humans [2]. Regardless of the remarkable progress which has been made in our understanding of dietary fat and obesity/type 2 diabetes (T2DM) with using HFD [3], it is important to point out that relatively little is currently available in regards to the study of HFDs and brain function and homeostasis, they have an obvious limitation in the study of brain dysfunction. Thus, HFD-induced models of metabolic dysfunction should be considered the best option for studying the effects of metabolic dysfunction on brain function.

The pathogenesis of the relationship of obesity, diabetes and neuropathy still far from observable, partly due to the lack of a suitable animal model that mimics human neuronal disease in T2DM, therefore we aimed to develop rodent model of obesity. There was a lack of records and research about the effect of obesity on brain pathophysiology and biochemical relationship. The probable for hydroxy citric acid (HCA) extraction of *G. Cambogia *to counteract the expected metabolic disturbance and brain dysfunction effects of obesity is important and merit investigation.

BChE is a serine hydrolase biochemically related to the cholinergic enzyme acetylcholinesterase. It is capable of hydrolyzing esters of choline. BChE has unique enzymatic properties and is widely distributed in the nervous system, raising the possibility of its involvement in neural function [4].

Adipose tissue has long been considered as a mere energy store but is increasingly appreciated for its roles in participating in several physiological and biochemical events such as inflammation, angiogenesis, hypertension, and vascular homeostasis. Moreover, the adipose tissue synthesizes and secretes a large number of factors collectively termed 'adipokines' which characterized for their role in influencing energy homeostasis and feeding behavior. However, an increasing number of studies have identified that adipokines may play a role in mediating long-term potentiation, neuroprotection, and neuroinflammation [5] and [6].

A major role for adipose tissue (adipocytes) is to sequester and store circulating lipid, which protects other cells and tissues in the body from the cytotoxic effects of free fatty acids in the circulation. Failure of adipocytes to effectively remove FFA from the circulation contributes to a variety of health complications including atherosclerosis, and ultimately the development of metabolic syndrome. Conversely, the deposition of adipose/lipid into muscle, liver, or bone marrow is known to promote not only metabolic syndrome but localized tissue dysfunction [7] which is the case with brain. Therefore, it is clear that adipocytes perform essential functions for the overall well being of individuals, but it is equally clear that adipose in the incorrect places or in excessive quantity also negatively impacts the health of an individual.

An increasing number of individuals regularly consume a diet high in fat, with HFD consumption known to be sufficient to promote metabolic dysfunction, although the links between high-fat diet consumption, brain oxidative and aging are now beginning to be elucidated.

Therefore the aim of this study was to develop rodent model of obesity by HFD and determine the metabolic disturbances compared to control diet and record the effect of obesity on brain and its effect on antioxidant defense system. Also biochemical changes in blood and tissue of rat and treatment of the affected group by *G. Cambogia*. Moreover, analysis of serum BChE activity in experimentally developed obesity and demonstrate the mechanisms that link obesity with altered brain function and propose future directions to be taken for addressing obesity oxidative stress and Alzheimer's disease.

## 2-Material and methods

### Material

#### 1- Diet

Two types of diets, control rat chow diet and special diet high in fat (35%) were used for induction of obesity in rats.

##### 1^st ^Normal *rat chow diet*

The standard normal rat chow consists of concentrate (350 gm), corn (600 gm), calcium carbonate, dicalcium phosphate, sodium chloride magnesium oxide and vitamins (50 gm). Standard normal rat diet composed of 65% carbohydrates (60% starch+5% sucrose), crude protein 20%, fat 5%, vitamins and minerals 5%, fibers 5%, metabolic energy of this diet is 2813 Kcal/Kg with 8% from fat.

##### 2^nd ^*the high fat diet*

HFD composed of 300 gm concentrates, 350 corn, 300 gm beef tallow, 50 gm vitamins, minerals and fibers according to (Kim et al. [8]. Calculations of high fat diet were 40% carbohydrate (starch 35%, 5% sucrose), 20% crude protein, 35% fat, 5% vitamins and minerals and fibers. Metabolic energy of this diet is 5130 Kcal/kg with 61% of this energy from fat.

#### 2- Experimental animals

The present study carried out on male Wister rat, (100-130 gm BW) 8 Wks age. They were purchased from Helwan town, Cairo, Egypt.

Rats were kept under observation for one week before onset of experiment to be acclimatized and housed individually in metal cages at room temperature 37°C, less than 12 hrs light/dark cycle in the laboratory of biochemistry in Faculty of Veterinary Medicine-Beni Suef University during the period of research. Water and special diet were allowed to rats in free access. Body weights were recorded weekly.

#### 3-Drugs for treatment

*G. Cambogia *is potent antiobesity drugs, the active principle is hydroxy citric acid (1,2-Dihydroxy-1, 2, 3-propanetricarboxylic acid) which used by dissolving one capsule in 10 ml D.W, each capsule containing 500 mg. *G. Cambogia *purchased from the EVA Pharm as a source of HCA.

Oral administration of 50 mg/rat daily dose was used according to the method described by Ohia et al. [9]. Handling of the animal was the same for all groups and did not affect weight gain. The drug was given to rat by developed stomach tube and given slowly to rat stomach.

### Methods

This experimental research was approved by the Committee of Scientific Ethics at Beni Suef University, faculty of veterinary medicine and consistent with its guidelines.

#### 1-Experimental design and animal grouping

Our experiments proceed for 12 week and include induction and treatment period and the whole number of rats was 24.

***Induction period ***began from 1^st ^to 8^th ^wk; it was the period of induction of obesity by feeding rats with HFD. The animals divided into 2 groups control and HFD groups. Control group, maintained on standard normal rat chow diet along the all period of experiment (12 wk) and include 8 rats, while HFD group was maintained on high fat diet. *Treatment period *start form 8-12^th ^wk during which HFD group continued on high fat diet and (HFD+G) treated group maintained on high fat diet to 4 wks (8 rats) with administration of *G. Cambogia *treatment at dose of 50 mg/rat/day by stomach tube.

##### Calculation of energy intake of consumed diet **and **body weight

Metabolic energy of standard rat chow and high fat diet were 2813 and 5130 Kcal/kg respectively. The average energy intake of consumed diet was calculating by multiplying the average consumed diet by 2.813 and 5.130 respectively according to consumed diet [10]. BW was measured every week.

#### 2 -Sampling and tissue preparation

##### Blood sampling

Blood samples were collected from medial canthus of the eye, via microhematocrit capillary tube at fasting state. The blood samples were collected in dry glass centrifuge tubes, allowed to coagulate at room temperature and centrifuged at 3500 rpm for 15 minutes for separation of serum. The clear, non-hemolysed supernatant sera were separated using clean dry disposable plastic syringes and stored at -20°C for subsequent biochemical measurements. Sodium fluoride used as anticoagulant for measuring of glucose in another blood sample.

##### Tissue sampling

At the end of experiment, rats were sacrificed by decapitation and abdominal incision was immediately done after taking of blood sample for obtaining of brain and abdominal adipose tissues. Brain was taken and washed by saline, dried by filter paper and homogenization of 0.5 gm of this tissue was done for tissue biochemical analysis of catalase, MDA and nitric oxide.

#### 3- Biochemical assays of serum and tissue

Serum was analyzed for total cholesterol, triglycerides and HDL levels. Also CK, γGT and α-amylase, BChE activity were measured colorimetrically using kits purchased from Bio-diagnostic (Cairo- Egypt). While blood uric acid and glucose level were estimated using Stanbio Laboratory USA Kits. Brain malondealdehyde (MDA) level was measured, as described by Mihara and Uchiyama [11], brain catalase (CAT) activity was determined according to the procedure of Cohen et al. [12] and brain NO according to Miranda et al., [13].

### Statistical analysis

Data were offered as mean ± SEM and were analyzed using one-way analysis of variance (ANOVA) followed by Tukey-Kramer methods for post-hoc analysis. A value of *p *< 0.05 was considered statistically significant. Graph Pad Prism 5 software (San Diego, CA, USA) was used for statistical analysis.

## 3. Results

### 3.1. Changes of BW, abdominal fat and energy intake during HFD and treatment

**Figure 1:**
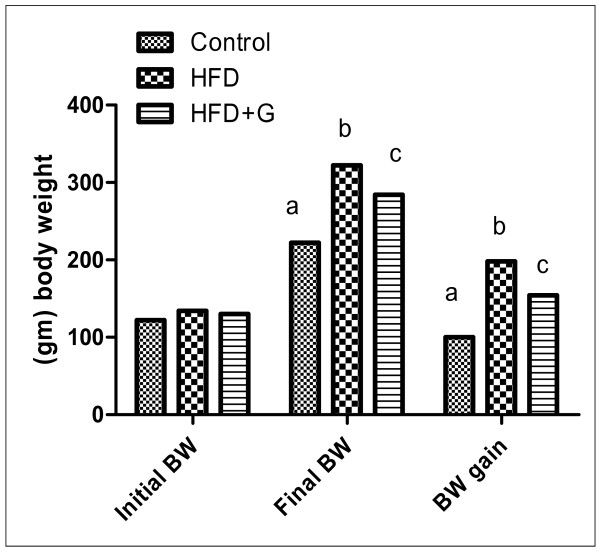
**Effect of initial, final and gain of body weight (gm) in the different groups of experiment**. In this study, final BW, BW gains (Figure 1) was significantly increased in HFD compared with control group. *G. Cambogia *treatment result in decrease BW, energy intake and adipose tissues in relation to HFD group.

**Figure 2:**
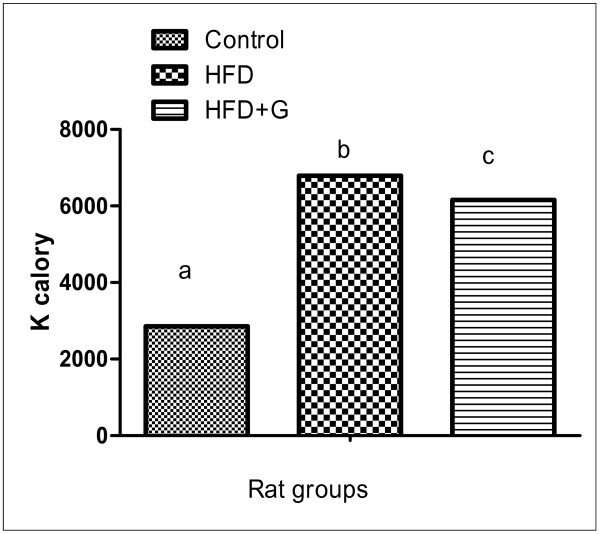
**Means of total energy intake k/calory in total period of experiment (3 months) in different groups of rats**. Figure (2) illustrated significant increase in energy intake in HFD compared with control. Values are statistically analyzed by one way ANOVA. Column have different letters indicate significant variation at (P ≤ 0.05), while the same letters indicate non significant variation.

In this study, final BW, BW gains (Figure 1) and energy intake (Figure 2) was significantly increased accompanied with significant increase in abdominal adipose tissue (Table 1) in HFD compared with control group. *G. Cambogia *treatment result in decrease BW, energy intake and adipose tissues in relation to HFD group.

**Table 1:** Changes in blood metabolic parameters CK, γGT, α-amylase, BCHE activity, Uric acid, abdominal fat and glucose level in control, HFD and HFD+G in rat.

*Groups Parameters*	Control	HFD	HFD+ G
**CK(U/L)**	70.0 ± 4.2^a^	63.0 ± 2.2^a^	69.0 ± 4.8^a^

**γGT (U/L)**	30.0 ± 2.7^a^	65.0 ± 6.2^b^	33.0 ± 3.5^a^

**α-amylase (U/L)**	93.0 ± 4.8^a^	150.0 ± 13^b^	110.0 ± 9.6^c^

**BCHE (U/L)**	260 ± 22^a^	500.0 ± 39^b^	300.0 ± 21^a^

**Uric Acid**	3.2 ± 0.13^a^	6.3 ± 0.32^b^	4.1 ± 0.16^a^

**Abdominal fat (g)**	4.98 ± 0.61^a^	9.2 ± 0.71^b^	5.0 ± 0.64^c^

**Glucose (mg/dl)**	59.0 ± 1.6^a^	111.0 ± 4.1^b^	91.0 ± 3.9^c^

Table (1) showed decreased activity of CK, while significant increase in γGT, α-amylase, BCHE activities. Moreover significant increase in abdominal adipose tissue, uric acid level increases significantly and hyperglycemia occur in HFD group relative to control. Some of these changes were significantly rearranged in HFD+G compared with HFD group ≤ 0.05.

Each value is the mean ± SEM. Means have different superscript letters indicate significant variation at (P ≤ 0.05), while the same letters indicate non significant variation

### 3.2. Effect of HFD and *G. Cambogia *treatments on metabolic parameters

Table (1) showed decrease in CK, while significant increase in γGT, α-amylase, BCHE activities. Moreover uric acid level increases significantly and hyperglycemia occurs in HFD group relative to control. Some of these changes were significantly rearranged in HFD+G compared with HFD group ≤ 0.05.

**Figure 3:**
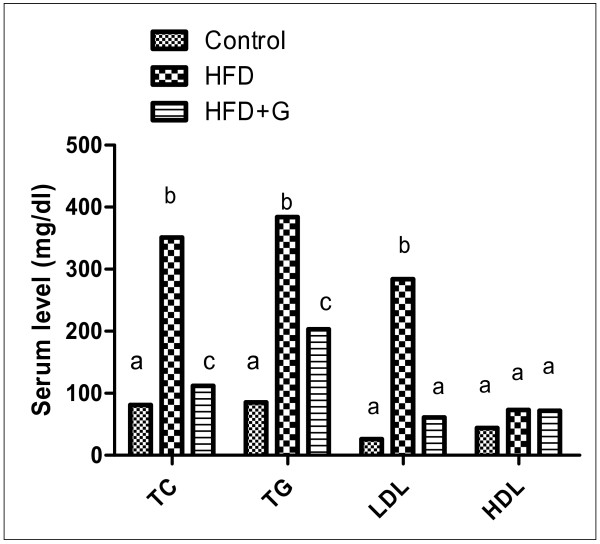
**Effect of lipid profile TC, TG, LDL and HDL (gm/dl) in the different groups of experiment**. Figure (3) demonstrated significant increase in serum lipid profile TC, TG and LDL level due to ingestion of HFD compared with control while, *G. Cambogia *extract improve these changes.

Figure (3) demonstrated significant increase in serum lipid profile TC, TG and LDL level due to ingestion of HFD compared with control and *G. Cambogia *extract improve these changes.

### 3.3. Effects of HFD and treatment on BOS (MDA and catalase activity)

In brain tissues, MDA level and catalase activities were significantly increased in HFD compared with control group and treatment with *G. Cambogia *ameliorated these changes (Table 2).

**Table 2:** Means of brain catalase, MDA and Nitric oxide levels in different groups of rats (3 months).

*Groups Parameters*	Control	HFD	HFD+G
**Brain Catalase (U/gm)**	5.1 ± 0.69^a^	12.2 ± 0.63^b^	10.3 ± 0.32^b^

**Brain MDA (nmol/g)**	2.6 ± 0.24a	5.7 ± 0.31^b^	4.9 ± 0.61^c^

**Brain Nitric oxide**	1.3 ± 0.08^a^	0.8 ± 0.11^b^	*1.1 ± 0.12*^*ab*^

In brain tissues, MDA level and catalase activities were significantly increased in HFD compared with control group and treatment with *G. Cambogia *ameliorated these changes (Table 2).

*Values are statically analyzed by one way ANOVA and reported as means ± SEM*.

Means have different letters indicate significant variation at (P ≤ 0.05), while the same letters indicate non significant variation

### 3.4. Correlation coefficient between parameters in different groups of rats

Significant positive correlation was detected between BChE activity and serum TG level (P < 0.03 and r = 0.96), TC (P < 0.001 and r = 0.99) and abdominal fat weight (P < 0.007 and r = 0.99), while serum BChE activity had a negative correlation with brain NO concentration (P < 0.04 and r = -0.96) (Table 3).

**Table 3:** Correlation coeffecient between parameters in different groups of rats.

	BChE	TG	TC	Glucose	Abdomin Fat	Final BW	Brain Catalase	Brain NO
**BChE**	----	P < 0.033 r = 0.96	P < 0.001 r = 0.99	NS	P < 0.007 r = 0. 99	NS	NS	P < 0.04 r = -0. 96

**TG**	-----	____	P < 0.048 r = 0.95	P < 0.02 r = 0.97	P < 0.021 r = 0.98	-------	P < 0.04 r = 0.90	P < 0.004 r = -0.99

**Brain MDA**	NS	P < 0.05 r = 0.95	NS	P < 0.007 r = 0.99	NS	P < 0.03 r = 0.91	P < 0.005 r = 0.97	P < 0.05 r = -0.95

**Brain Catalase**	NS	-----	NS	P < 0.006 r = 0.99	NS	P < 0.03 r = 0.91	____	P < 0.04 r = -0.95

**Brain NO**	------	------	P < 0.05 r = -0.94	P < 0.024 r = -0.97	P < 0.02 r = -0.98	NS	P < 0.05 r = -0.94	____

**Final BW**	NS	P < 0.008 r = 0.99	NS	P < 0.013 r = 0.98	P < 0.035 r = 0.96	-----	P < 0.03 r = 0.97	P < 0.002 r = -0. 99

*r = Value of correlation coefficient. P *≤ *0.05 indicate significant variation*.

Significant positive correlation was detected between BChE activity and serum TG level (P < 0.03 and r = 0.96), TC (P < 0.001 and r = 0.99) and abdominal fat weight (P < 0.007 and r = 0.99), while serum BChE activity had a negative correlation with Brain NO concentration (P < 0.04 and r = -0.96) (Table 3).

Brain MDA positively correlated with TG (P < 0.05 and r = 0.95), blood glucose (P < 0.007 and r = 0.99) and final BW (P < 0.03 and r = 0.91) while, negatively correlated with NO (P < 0.05 and r = -0.95). There were negative correlation between blood glucose and brain NO (P < 0.024 and r = -0.9) (Table 3) in rats group. Also the data showed negative correlation between brain NO and MDA levels (P = 0.05 and r = -0.95), (Table 3).

Brain MDA positively correlated with TG (P < 0.05 and r = 0.95), blood glucose (P < 0.007 and r = 0.99), final BW (P < 0.03 and r = 0.91) and brain catalase (P < 0.005 and r = 0.97) while, negatively correlated with brain NO (P < 0.05 and r = -0.95). There were negative correlation between blood glucose and brain NO (P < 0.024 and r = -0.9) (Table 3) in rats group. Also the data showed negative correlation between brain NO and MDA levels (P = 0.05 and r = -0.95) (Table 3).

## 4-Discussion

### 4-1- Role of HFD and treatment in BW and abdominal fat

Our data showed that there were significant increase in BW and abdominal fat in HFD compared to control group (Figure 1 and table 1). This may due to increase feed and caloric intake.

Consumption of the *G. Cambogia *extract effectively lowered the BW, visceral fat accumulation and improving dyslipidemia in HFD group, probably by modulating multiple genes associated with adipogenesis, such as adipocyte protein aP2 and sterol regulatory element-binding factor [8].

HCA, an active constituent of the *G. Cambogia*, diminish food utilization in rodent models of obesity may be by diverting carbohydrates and fatty acids that would have become fat in the liver into hepatic glycogen. This metabolic change may send a signal to the brain that result in a reduced appetite, so reduce BW which may be due to HCA combined effects on the metabolic and serotonin pathways [14].

We can conclude that increase feed intake due to HFD significantly amplify body weight and abdominal fat while treatment with *G Cambogia *decline BW and abdominal fat.

### 4.2. Metabolic disturbance of dyslipidemia and brain dysfunction in HFD and treatment groups

Currently there are believed to be 41 million Americans that can be considered to be prediabetic, with the prediabetic state associated with a progressive development of multiple metabolic disturbances which precedes the irreversible stage of diabetes and importantly can even occur independent of the development of diabetes [15].

The current results indicated a significant increases in serum lipid profile (TC, TG and LDL levels) in HFD compared to control (Figure 3) resulting in dyslipidemia which is a common disturbance associated with HFD consumption.

The link between dyslipidemia and having a higher risk of cognitive impairment or dementia is controversial. Reduced levels of high-density lipoprotein cholesterol have been reported in some cases of dementia but not all cases of dementia [16].

Concerning records for HFD exacerbating brain the current experiment showed significant increases in energy intake in HFD compared to control diet (Figure 2). Moreover rats fed HFD had both hyperglycemia and hypertriglyceridemia. Various forms of dietetic fat differentially change the expression of neuropeptide genes involved in energy homeostasis [17].

HFD consumption is thought to induce changes not only in energy metabolism but also in brain function which may be due to disturbances of ATP in brain cells as the main cause of various brain disorders. HFD consumption and the presence of metabolic dysfunction are sufficient to exacerbate brain aging, promoting the development of cognitive alterations including dementia [18]. Moreover, fatty acid overload or metabolic dysfunction might impair neural control of energy homeostasis and contribute to obesity and/or T2DM in predisposed subjects [19].

Biochemically, HFD-induced disturbances in metabolism resulting in increase in the flux of free fatty acids (FFA) into a variety of tissues (liver, pancreas and muscle), which is subsequently followed by the deposition of triglycerides into these tissues. Elevations in FFA promote metabolic disturbances by impairing the ability of cells to store glucose as glycogen and to respond to insulin, which cumulatively are capable of promoting the development of diabetes. Cumulatively, these events lead to hyperinsulinemia and hyperglycemia (Table 1) due to impaired insulin-mediated regulation of glucose levels.

HFD consumption probably mediates brain disturbances may be via reducing the ability of the brain to successfully respond to stressors (as is observed in other tissues). A decreased capacity to repair or replace damaged brain system may play a causal role in promoting both neuropathology and neurobehavioral deficits [20].

For example, decreased trophic factor support or vascular support in response to a western diet as reported in non-CNS tissues would be expected to potentially contribute to the development of neuropathology and dementia.

Multiple metabolic defects in T2DM directly injure dorsal root ganglia neurons through different mechanisms that all result in oxidative stress. Dyslipidemia leads to high levels of oxLDLs that may injure dorsal root ganglia neurons via LOX-1 and contribute to the development of diabetic neuropathy [21].

The indication for HFD promoting brain pathogenesis could be summarized as; HFD-induced metabolic dysfunction promotes cognitive alterations and dementia [22].

Studies carried out in rats in agreement with our biochemical evidence, have shown that, a diet high in saturated fatty acids can impair learning and memory functions. In addition, the strong relationship between obesity and cognitive impairment found in animals [23].

The serum concentrations of cholesterol and triglyceride were significantly lower in animals that were given the *G. Cambogia *than in the HFD control group. These results may attribute to the effect of HCA that inhibit the lipogenesis catalyzed by ATP citrate-lyase in the liver and peripheral tissues; also it prevents the production of acetyl-CoA and, subsequently, malonyl-CoA [24].

### 4.3. Changes of CK, amylase, γGT, uric acid and glucose as indicators for metabolic disturbances in HFD and treatment groups

Activity of creatine kinase (Table 1) was decreased while an increase in lipid peroxidation (MDA) was observed in HFD compared to control group.

Creatine kinase plays a key role in energy metabolism of nervous tissue and might be one of the targets for reactive oxygen species (ROS) [25]. HFD was shown to produce an increase of serum CK activity in rats [26]. Moreover obesity is associated with an increase in the percentage of fast twitch, oxidative-glycolytic muscle fibers that use glucose as an energy substrate. Therefore, an increase of CK under HFD conditions may reflect an increase of glycolytic enzyme activity.

Pancreatic α-amylase level was reduced in HFD compared to control (Table 1). We suggest that the biochemical assay of pancreatic enzymes might be of value in determining the severity of obesity and could be used as a parameter in evaluating the response to treatment [27].

γGT activity was higher in HFD compared with controls (Table 1) this result associated with metabolic abnormalities and correlated with metabolic syndrome individual components including, hyperglycemia and insulin resistance [28]. Xu et al., [29] recorded that there was significant and independent associations of γGT and ALT with metabolic syndrome and there are positive associations between serum γGT and other concurrent metabolic abnormalities which resulting in brain dysfunction.

Risk for the metabolic disturbances components increased as the baseline γGT activity increased, indicating that high γGT activity predicted future development of metabolic disturbances.

Uric acid has been known to exert neuroprotective effects by acting as a free radical scavenger; however, several observational studies indicated that high levels of serum uric acid increased the risk of cardiovascular events or stroke. It was sought to determine whether increased levels of uric acid are associated with the presence of silent brain infarction (SBI) [30]. The current data showed elevation in uric acid in HFD associated with hypertriglyceridemia and hypercholesterolemia suggesting that an increased level of uric acid may be a risk factor for the presence of SBI. So serum uric acid level might be a good serum marker of underlying SBI or future stroke.

High levels of urate have been associated with diabetes, with increased burden of cerebral ischemic pathology, increased risk of mild cognitive dysfunction and ischemic white matter lesions, which are risk factors for cognitive impairment [31].

Impaired antioxidant defenses are implicated in neurodegenerative disease. The blood levels of uric acid, a water-soluble antioxidant, are elevated in HFD may be to compensate the oxidative stress that may occur and act as response to the metabolic disturbances and its level may be modified by the HFD.

Recently Wu et al., [32] study the association of hyperuricemia (HUA) with metabolic disorders and identify the risk factors of HUA and concluded a several risk factors contribute to the occurrence of elevated serum uric acid in T2DM, and metabolic disorders and complications are also closely associated with HUA. Also patients with HUA had significantly higher incidences of carotid atherosclerosis, cerebral infarction and diabetic nephropathy than those with normal uric acid level.

Insulin has been suggested to play important roles in regulating memory, so any disturbance in insulin signaling would be expected to have a negative impact on memory [33]. Some studies have demonstrated that HFDs were able to increase amyloid beta peptide (Ab) levels and Ab-neuropathy in brains of mice exhibiting Alzheimer's disease pathology.

Recent studies suggested that hyperinsulinemia and insulin resistance are linked to Alzheimer's disease (AD) and the increased amyloid beta-peptide (Abeta) in the brain of HFD-fed mice is associated with reduced brain-derived neurotrophic factor expression, which led to abnormal feeding behavior and increased food intake, resulting in obesity and insulin resistance in these animals, so experimental evidence suggests that alterations in glucose and insulin metabolism may influence the onset of AD through their influence on the synthesis and degradation of Ab peptides [34].

One possible mechanism by which obesity may increase the risk of stroke is via an alteration in cerebral perfusion. Cerebral perfusion is regulated through active constrictor and dilator mechanisms and by the physical properties of the cerebral vasculature.

The changes of biochemical markers of obesity as abdominal obesity, high BW and high caloric intake (Figure 1, 2) associated with hypertriglyceridemia, hypercholesterolemia, high LDL (Figure 3), high γGT, hyperuricemia and hyperglycemia (Table 1), representing metabolic disturbances that consider a pre-diabetic state which spot that harmful changes in metabolism can predispose and predict T2DM.

### 4-4- Role of BChE activity in HFD and treatment groups

A significant increase in serum BChE activity in HFD was observed when compared with the control group (Table 1). These findings agree with recent studies which revealed elevated serum and tissue BChE actvity in obesity, T2DM and Alzheimer's disease. Acetylcholine (ACh) has antiinflammatory action; hence elevated BChE resulted in reduction in acetylcholine level that could trigger the onset of low grade of systemic inflammation seen in T2DM and Alzheimer's disease [35] and [36].

Significant positive correlation was detected between BChE activity and serum triglyceride levels (P < 0.03 and r = 0.96), TC (P < 0.001 and r = 0.99) and indicators of abdominal fat obesity (P < 0.007 and r = 0.99), while serum BChE activity had a negative correlation with brain NO concentration (P < 0.04 and r = -0.96) (Table 3).

These findings are important predictors of BChE activity and suggested an involvement of BChE in the pathophysiological process constituting the metabolic syndrome. Whether elevated BChE activity precedes or follows the development of risk factors is not known.

The opinion that obesity influence CNS had been raised and there are link between adiposity and increased risk of Alzheimer disease. Moreover there were relation between insulin resistance, adiposity and Alzheimer due to insulin advanced product of glycosylation, cerebral vascular disease and product of adipose tissue [37].

Serum activity of the BChE are affected by dietary fat, obesity, hyperlipidemia and diabetes mellitus. Similarly, diabetes mellitus has been shown to alter the levels of the enzyme. BchE, which can affect cell membrane oxidative stress and fluidity, is structurally homologous to lipase [38]. However other studies have suggested BchE may not have a direct pathophysiological role in the development of metabolic syndrome, but may be considered as secondary markers for this syndrome in obese individuals [39].

In consonance with other studies, BChE activity was related to changes in serum lipids profile; however there was a relationship to serum triglycerides and serum cholesterol in our study. BChE activity could also be used to select the drug for treatment of hypertriglyceridemia in T2DM. Similarly diabetes was one of the risk factors for coronary artery disease that independently correlated with BchE activity [36].

High BChE activity in overweight rats, decrease the vasodialting effect of acetylcholine via its hydrolysis and associated with lowering the brain NO level causing vasoconstriction of brain blood vessels rendering it susceptible to dysfunction. So this is the first and a novel data that explore the role of BChE activity and brain NO associated with oxidative stress in brain dysfunction of long term HFD ingestion.

The effects of *G. Cambogia *on impaired ACh-stimulated vasodilation through restoring the brain NO level and decrease brain catalase and MDA compared to the HFD support the hypothesis that oxidative stress contributes to brain endothelial dysfunction in obesity.

In particular, recent confirmation indicates that along with acetylcholinesterase, BChE catalyzes the hydrolysis of acetylcholine, and thus serves as a co-regulator of cholinergic transmission. BChE is likely to be involved in neurodegenerative disorders such as Alzheimer's disease [4]. Therefore, inhibition of BChE by using *G. Cambogia *(Table 1) will not only produce better cholinergic transmission but also has the potential to interfere with the disease process in Alzheimer's disease and other dementing disorders. This change raises the chance of the possible use of BChE inhibitors for the treatment for obesity and associated disorders.

There were normalization in nervous system which indicated by inhibition of BChE activity in HFD+G compared with HFD group (Table 1). This may be because of super citrate max (HCA-SX) attenuate the increase in oxidative stress by decreasing lipid peroxidation and declining oxidative stress biomarkers in tissues [40].

It could be concluded that serum BChE activity is increased in experimentally overweight rats and is associated with biochemical parameters of adiposity while, *G. Cambogia *ameliorated these changes.

### 4.5. Role of HFD and treatment on brain tissues oxidative markers (catalase and MDA) in rats

In our experiment there was a significant increase in brain catalase activity in HFD relative to control group (Table 2). This change result from activation of biochemical pathway leading to increased level of ROS and amplified lipid peroxidation producing oxidative stress. It appears that the modulation of antioxidant enzyme activity plays a significant role in the initial elevations in oxidative damage following HFD consumption.

The increment of brain catalase activity give evidence for the relation between obesity, T2DM and oxidative stress which agree with Houstis et al. [41] who reported that insulin resistance accompanied by oxidative stress indicated by increase ROS, moreover there are increased superoxide potential, NADPH oxidase in obese patient. Obesity result in metabolic syndrome this syndrome (e.g T2DM) results form activation of biochemical pathway leading to increased level of ROS and increased lipid peroxidation generate oxidative stress.

Our data demonstrate that consumption of HFD for 12 weeks was adequate to increase the level of MDA as marker of oxidative stress in the brains of rats (Table 2). This change accompanied with significant hypercholesterolemia and hypertriglyceridemia which may be due to HFD ingestion.

Brain MDA increased with obesity and positively correlated with TG (P < 0.05 and r = 0.95), blood glucose (P < 0.007 and r = 0.99) and final BW (P < 0.03 and r = 0.91) while, negatively correlated with NO (P < 0.05 and r = -0.95), (Table 3) where MDA represented as indicator for undesirable lipid peroxidation. Moreover, TG positively correlated significantly with brain MDA, Catalase as indicator of oxidative stress, suggesting association between lipid peroxidation and brain biochemical alterations.

Oxidative stress and brain inflammation are thought to play key roles in HFD-induced cognitive loss, as it have been found to be increased in mice fed HFD which show clear cognitive impairment [42].

There is increasing alertness of the everywhere role of oxidative stress in neurobehavioral toxicity [43]. The brain is vulnerable to oxidative stress damage because of its high energy utilization, low levels of endogenous scavengers (as vitamin C, catalase and superoxide dismutase), high metabolic load, wide axonal and dendritic networks, and high content of lipids, polyunsaturated fatty acids and proteins [44]. Also the brain is a highly vascularized renders it, extremely susceptible to undergo rapid elevations in oxidative damage.

Consumption of HFD may promote lipid peroxidation in the brain by elevating the levels of ROS, as has been found in non-CNS tissues following consumption of a HFD [26]. While the initial products of polyunsaturated fatty acid oxidation are almost exclusively monohydroperoxides, these products are often rapidly put through a progression of secondary and tertiary oxidative modifications that involve the genesis of lipid peroxyl and lipid alkoxyl radicals. These increasingly toxic species are capable of rapidly inducing a milieu of deleterious signaling cascades, promoting disturbances in the plasma membranes and inactivating vital cellular enzymatic activities.

However, because the whole brain tissue was utilized in the current study, we may have missed the presence of brain region specific elevations in oxidative stress. For example, previous studies have demonstrated that a 4 month diet of a highly palatable diet selectively increases oxidative stress in the rat frontal cortex [45]. Regardless, the presence of elevated lipid peroxidation in this study suggests that oxidative stress, via the elevation of potentially toxic products such as lipid hydroperoxides, may promote cellular stress and toxicity in the brain following even the short-term consumption of a WD or HFD.

Studzinski et al., [46] found that short-term WD is sufficient to selectively promote cerebral oxidative stress and metabolic disturbances in mice, with increased oxidative stress preceding alterations in Abeta. These data have comparable and agreement to ours and have important implications for understanding how WD or HFD may potentially contribute to brain dysfunction and the development of neurodegenerative disorders such as Alzheimer's disease.

However, the role of ROS in the brain has not been elucidated, although high dietary fat has been reported to activate NADPH oxidase-derived increased oxidative stress and inflammation in the brain [47]. Our viewpoint that oxidative stress in the brain exerts an effect on central sympathoexcitation in HFD-induced obesity is plausible because recent studies have also implicated the contribution of ROS overproduction in the brain for activation of the sympathetic nervous system.

The current study raises the possibility of hyperglycemia contributing to brain pathogenesis and altered brain function via the generation of BOS. Similarly, other data raise the possibility that circulating levels of adipokines may become altered in models relevant to AD, with a neuroprotective role for adipokines emerging in a number of paradigms relevant to AD [48].

Taken together, our studies raise the possibility of diet contributing to brain pathogenesis, via the generation of cerebral oxidative stress. The current data have important implications for understanding how diet and oxidative stress may ultimately contribute to the development of diet-related neurodegenerative disorders. We recommended that BOS is an important initial factor for the progression of brain pathology, with oxidative stress potentially preceding the development of AD.

Clarifying the cellular mechanisms responsible for increased levels of oxidative stress and identifying the peripheral factors by which diet modulates cerebral oxidative stress, will likely be a key to the development of effective interventions to reverse or prevent the deleterious effects of a HFD on the brain.

There were significant decreased in level of MDA in HFD+G group (Table 2) compared with HFD group as result of (-)hydroxy citric acid inhibit ATP citrate lyase which catalyse extramitochondrial cleavage of citrate to oxaloacetate to acetyl COA which used in fatty acid synthesis and TC and TG synthesis. Moreover *G. Cambogia *(HCA) has hypolipidemic action and there was marked reduction in MDA brain which was due to effect of *G. Cambogia *that reduce MDA in tissues which was recorded by Asghar et al. [40] indicating that HCA improve lipid peroxidation.

It become noticeable that reduction of energy intake decrease oxidative stress and diminish metabolic syndrome. In this concern *G*. Cambogia administration affect on feed intake and appetite by increasing availability of 5 hydroxy tryptamine and enhance serotonin in brain that reduce appetite and inhibiting body fat biosynthesis [9]. Moreover super citrate max (HCA-SX) attenuates the increase in oxidative stress [40]. These findings consistent with our data where there were reduction in catalase activity and significant MDA level in HFD+G compared with HFD group indicating the antioxidant effect of *G. Cambogia*.

It could be conclude that feeding HFD to rats increases level of lipid profiles which consequently increase the level of BOS and as a tissue toxicant accompanied with increased activity of γGT, α-amylase, BChE, uric acids. While using of *G. Cambogia *that contain hydroxyl citric acid as antiobesity agent decrease TC, LDL which directly affect MDA levels and improving brain dysfunction.

### 4-6- Role of diet induced obesity & treatment on brain tissues NO level

Our data showed a significance decrease in NO brain level in HFD (Table 2) compared with control group as result of obesity and hypertriglyceridemia, this decrement may be due to decreased bioavailability of NO and reduce signaling of NO [49] that indicated by reduced NO metabolite (nitrite and nitrate) production within the brain tissue in HFD.

Obesity associated with hyperglycemia is a key factor that contributes to the development of diabetes-related microvascular disease, cyclooxygenase which lead to increased oxidative stress and reduction in generation of nitric oxide in microvessels endothelial cells [50].

Nitric oxide signaling involved in the regulation of food intake and insulin signaling, is altered in obesity and diabetes. Hyperglycemia impairs glucose and insulin regulation of NO production where glucose inhibits NO production which may occur through AMP-activated protein kinase inhibition in ventromedial hypothalamus neuron [51]. These findings supported by negative correlation between glucose and brain NO (P < 0.024 and r = -0.9) in HFD group (Table 3).

We found NO inactivation by ROS and functional NO deficiency in our model which indicated by negative correlation between NO and MDA oxidative stress in brain tissues (P < 0.05 and r = -0.95) (Table 3). Also enhanced ROS-mediated inactivation and sequestration of NO, which may contribute to the reduction of bioactive NO and dysregulation of NO synthesis isotype [52], given the critical role of NO in BOS and function.

Concerning the relation of NO and BOS, a few researchers showed that exposure of rats to restraint stress suppressed behavioral activity in the elevated plus maze and this was associated with increases in brain MDA and decrease in reduced glutathione and brain NO metabolite levels in brain homogenates [53]. Moreover, Gulati et al., [54] reported that L-Arginine pretreatment was more effective in modulating the restraint stress induced stress responses/markers in the high emotional group of rats and suggest that NO plays a differential role during exposure to acute and repeated stress situations, and that the relationship between stress and emotionality status may be under the regulatory influence of NO.

Decrement of brain NO level in HFD group, initiate vasoconstriction that affect brain and using methods for controlling obesity suppose to increase NO level and improving state of different organs that apparent in our results due to action of nitric oxide as vasodilator. This study provide persuasive evidence for the presence of brain endothelial dysfunction in obesity which associated with high abdominal fat, hyperglycemia, hypertriglyceridemia, hypercholesterolemia, abnormalities of LDL, high BChE, γGT activity and brain oxidative stress as a potential factors leading to endothelial dysfunction.

The current biochemical data (Table 2) suggest that treatment with *G. Cambogia *has a protective effect on oxidative stress induced alteration of brain oxidative injury in HFD-rats and NO may be involved in this effect. In accordance with experimental data we suggested that the main signal molecule causing changes in enzyme activity should be NO. NO synthetase activity from the HFD group was decreased associated with diminished L-arginine transport [55], consequently *G. Cambogia *decrease TG leading to replenish the level of NO to normal.

The possibility that HFD associated with metabolic disturbance and brain risk is alarming given international trends of overweight and obesity in the general population. However, prevention and manipulation of adiposity may also provide a means to prevent AD.

Our data offer novel insights into potential mechanisms of brain dysfunction in obesity and the BChE and γGT inhibition and NO elevation might represent a novel strategy for the treatment of obesity with dyslipidemia and BOS, also demonstrate the efficacy of HCA in weight management.

## Conclusion

We could concluded that the mechanism by which HFD could affect brain dysfunction is by rising obesity indicators, lipid profile (TG, TC, LDL) and disturbing the metabolic parameters (CK, γGT, uric acid and glucose in blood) that correlated with BChE and associated with BOS marker (brain MDA and catalase) and reduction of brain NO resulting in brain lipid peroxidation, cerebrovascular changes and consequently dysfunction and susceptible to disease as AD while *G. Cambogia *as antiobesity and antioxidant agent rearrange these changes.

## Competing interests

The authors declare that they have no competing interests.

## Authors' contributions

KA, HK and MA carried out experimental work; biochemical analysis, interpretation and discussion of the results related to their part of the work. KA performs the design and planning of the study, statistical analysis, wrote the paper, drafting and revision of the manuscript. All authors read and approved the final manuscript.
